# A Standardized Nomenclature Design for Systematic Referencing and Identification of Animal Cellular Material

**DOI:** 10.3390/ani14111541

**Published:** 2024-05-23

**Authors:** Lisa Schrade, Nancy Mah, Anita Bandrowski, Ying Chen, Johannes Dewender, Sebastian Diecke, Christian Hiepen, Madeline A. Lancaster, Tomas Marques-Bonet, Sira Martinez, Sabine C. Mueller, Christopher Navara, Alessandro Prigione, Stefanie Seltmann, Jaroslaw Sochacki, Magdalena A. Sutcliffe, Vera Zywitza, Thomas B. Hildebrandt, Andreas Kurtz

**Affiliations:** 1 Fraunhofer Institute for Biomedical Engineering (IBMT), 66280 Sulzbach, Germany; 2 Department of Reproduction Management, Leibniz Institute for Zoo and Wildlife Research (IZW), 10315 Berlin, Germany; 3 Department of Neuroscience, FAIR Data Informatics Lab, University of California San Diego, San Diego, CA 92093, USA; 4 SciCrunch Inc., San Diego, CA 92192, USA; 5 Technology Platform Pluripotent Stem Cells, Max Delbrück Center for Molecular Medicine in the Helmholtz Association (MDC), 13125 Berlin, Germany; 6 MRC Laboratory of Molecular Biology, Cambridge Biomedical Campus, Cambridge CB2 0QH, UK; 7 Institute of Evolutionary Biology, Pompeu Fabra University—Spanish National Research Council, ICREA, 08003 Barcelona, Spain; 8 Catalan Institution for Research and Advanced Studies (ICREA), 08010 Barcelona, Spain; 9 Centro Nacional de Analisis Genomico (CNAG), 08028 Barcelona, Spain; 10 Catalan Institute of Palaeontology Miquel Crusafont, Universitat Autònoma de Barcelona, 08193 Barcelona, Spain; 11 European Molecular Biology Laboratory (EMBL) Barcelona, 08003 Barcelona, Spain; 12 San Antonio Cellular Therapeutics Institute, University of Texas at San Antonio, San Antonio, TX 78249, USA; 13 Department of General Pediatrics, Neonatology and Pediatric Cardiology, Duesseldorf University Hospital, Medical Faculty, Heinrich Heine University, 40225 Duesseldorf, Germany; 14 Faculty of Veterinary Medicine, Free University of Berlin, 14163 Berlin, Germany; 15 Berlin Institute of Health (BIH), Center for Regenerative Therapies (BCRT), 13353 Berlin, Germany

**Keywords:** viable cell material, wildlife, biosample nomenclature, cryobanking, standardization, unambiguous identifier, FAIR data, biodiversity, species conservation

## Abstract

**Simple Summary:**

A permanent link between biological samples and the associated data is essential for their effective and long-term utilization. In order to enable clear identification and referencing of biosamples and to ensure comparability in research, explicit naming of such material by assigning unique and permanent identifiers is therefore necessary. This can be achieved by using explicit naming structures with a predefined pattern. These nomenclature structures have been developed for diverse biological materials but are lacking for animal cellular material, such as tissues and cell lines. Here, we present a first, standardized, human-readable nomenclature design, which generates clear and stable identifier names for such material with a focus on cellular material from wildlife species. Consistent application and central distribution and storage of these identifiers are required to ensure explicit identification and traceability of animal biosamples. This novel and globally applicable identification system adds standardization to the long-term storage of animal cell material in cryobanks and supports species conservation and research.

**Abstract:**

The documentation, preservation and rescue of biological diversity increasingly uses living biological samples. Persistent associations between species, biosamples, such as tissues and cell lines, and the accompanying data are indispensable for using, exchanging and benefiting from these valuable materials. Explicit authentication of such biosamples by assigning unique and robust identifiers is therefore required to allow for unambiguous referencing, avoid identification conflicts and maintain reproducibility in research. A predefined nomenclature based on uniform rules would facilitate this process. However, such a nomenclature is currently lacking for animal biological material. We here present a first, standardized, human-readable nomenclature design, which is sufficient to generate unique and stable identifying names for animal cellular material with a focus on wildlife species. A species-specific human- and machine-readable syntax is included in the proposed standard naming scheme, allowing for the traceability of donated material and cultured cells, as well as data FAIRification. Only when it is consistently applied in the public domain, as publications and inter-institutional samples and data are exchanged, distributed and stored centrally, can the risks of misidentification and loss of traceability be mitigated. This innovative globally applicable identification system provides a standard for a sustainable structure for the long-term storage of animal bio-samples in cryobanks and hence facilitates current as well as future species conservation and biomedical research.

## 1. Introduction

### 1.1. Background

As biodiversity is increasingly threatened, studying it, with aims to its conservation and restoration, is needed. Besides nature conservation efforts, the collection and active preservation of living biological samples usable for research (e.g., for the generation of germ cells) also plays an increasingly crucial role in species conservation [1]. The global exchange of such rare and valuable biosamples for research and species conservation requires traceability [2]. Standardized naming tools for generating unique identifiers (UIs) support such mandatory tracing, enable clear referencing and have been broadly discussed for human cells [3,4], human genomic data [5] and human gene products [6,7]. A widely used naming tool for human pluripotent stem cells was proposed by Luong et al. (2011) [3], further developed by Kurtz et al. (2018) [4] and implemented in the Human Pluripotent Stem Cell Registry (hPSCreg) [8]. This is the only available nomenclature specific to human pluripotent stem cells suitable for generating human-readable, i.e., interpretable UIs [9,10]. 

For referencing of a specific entity, identifiers serve as a link with which metadata are associated. Multiple identifiers may co-exist to complement their particular features and utility, such as information content, coding length and global uniqueness. However, if different identifiers exist for one entity, they should be unambiguously linked and reference one another, e.g., to connect data repositories [11]. The Resource Identification Initiative (RII) [12] introduced the concept of Research Resource Identifiers (RRIDs) [13] to enable reproducible research through the use of RRIDs, unique alphanumerical identifiers for referencing published research materials, such as reagents, tools, organisms and biological materials. For vertebrate and invertebrate cell lines cited in the scientific literature, Cellosaurus’ knowledge resource [14] assigns a short, persistent, unique stable identifier, which is recognized as the RRID of these cell lines [15]. Furthermore, the BioSamples database at the European Bioinformatics Institute as part of the European Molecular Biology Laboratory (EMBL-EBI) [16] assigns unique accession numbers to registered research biosamples, including living cells and tissues of all kinds of human and non-human organisms used for sequencing [17,18]. However, without disclosure of the linked metadata, the mutually independent RRIDs and BioSamples identifiers are not directly informative or human-readable. Nor do they allow for the assessment of kinships or complex relationships between origin, donor species, biosample type and derivative, which are needed for many application cases of animal cells. Consequently, any newly established biosample identifier should fulfill the specific stakeholder need for human readability but also establish and maintain stable links to a respective RRID to enable traceability. A central platform is required to issue and register human-readable names directly attributed to the relevant data and recorded with the RRID authorities so that research resources can continue to be resolved by their RRIDs. In conclusion, no uniform, human-readable, informative nomenclature exists for animal living biosamples such as tissues, derived cell lines, gametes or embryos to enable traceability to its origins and legal provenance.

Research with living animal biosamples, especially cell lines, is the focus of various scientific fields, such as species conservation, basic research and comparative biological research, as well as veterinary and biomedical research [19], and the derivation and establishment of new animal cell lines are expanding [20]. Further, the publication of animal cell lines in general and derived pluripotent animal stem cell lines in particular (i.e., embryonic stem cells (ESCs) and induced pluripotent stem cells (iPSCs)) is constantly increasing. This applies to wildlife species threatened with extinction [21,22,23,24,25,26], as well as domesticated and livestock species [27,28,29,30,31,32,33] and model species, including non-human primates, mice, naked mole rats and others [34,35,36,37,38,39]. Also, the establishment of stem-cell-derived multicellular models such as organoids, assembloids and blastoids [40,41] is progressing for animal species, and they have been published both for model species, e.g., for mouse blastoids [42], and wildlife species, e.g., for rhinoceros cerebral organoids [26]. Research on the cellular material of domesticated model species has been conducted intensively in the last decades, with the result being more than 174,000 mouse (*Mus musculus*) ES cell lines having been established, registered by the RII and assigned an RRID so far [43] and more than 2 million mouse biosamples having been registered in the BioSamples database. In contrast, living cellular biosamples of wildlife, i.e., non-domesticated species [44], have been less strongly researched yet are steadily growing in number. As a result, animal biosamples are increasingly exchanged and processed by different research institutions worldwide [2]. This demands unambiguous identification to assure access to and the traceability and easy authentication of samples and cells.

### 1.2. Need for a Standardized Nomenclature Design for Animal Biosamples

#### 1.2.1. Free-Text Names Have Little or No Interpretability

The absence of a standardized naming system at present has led to a wide range of inconsistent naming structures for animal cellular material (see Table 1). These irregular name schemes range from purely descriptive, alphabetical styles to short alphabetical and alphanumerical names and further to long alphanumerical names with or without additional structuring characters (see examples 1–5 in Table 1). The inconsistency in the naming of animal cellular material and the subsequent irregular interpretability can be illustrated by examples for fibroblast cell lines such as “ENL-2” and “KCB 96008”, both from Asian elephants (*Elephas maximus*); “KDF” and “SR-fibroblasts”, both from Sumatran rhinoceroses (*Dicerorhinus sumatrensis*) and “Fish 80” and “pA03_wD06”, both describing tissue samples of rainbow trout (*Oncorhynchus mykiss*). Moreover, allocated cell line names such as “UCLAi090-A” mimic and can be confused with published naming structures intended for human cell lines (see examples 6–12 in Table 1). If publicly accessible, most of these biosamples are assigned an RRID (“CVCL_xxxx”) or BioSample ID (“SAMxxxxxxxxx”) characterized by unique alphanumerical coding (“xxxxxx”). Thus, the identifier is not informative about any features of the biosample. These examples show existing ambiguities in the naming of biosamples and clearly demonstrate the necessity for a uniform nomenclature and informative identifier system in research with animal cells.

#### 1.2.2. Scientific Exchange and Cryobanking

The distribution of animal biosamples, such as tissues, cells and gametes, between research labs and other resources, for example, cryobanks, without a persistent standard identification system impedes explicit referencing and traceability, particularly when cells are modified, such as, for example, by reprogramming them into iPSCs. Induced pluripotent stem cell lines are immortal, making them valuable tools for differentiation and further genetic modification [34]. Data related to the cell material, such as information on its derivation, cultivation and characterization, as well as ethical and legal provenance, are at risk of being disassociated from the cells over time and their ease of global distribution hindered [3,4]. In addition, inconsistent naming complicates conducting literature searches for existing cell lines, hampers the findability, accessibility, interoperability and reuse (FAIR) principles [11,55] and increases the chances of gross misidentification of cell material and the subsequent irreproducibility of published results [56].

The international exchange and utilization of non-human genetic resources (i.e., “to conduct research and development on the genetic and/or biochemical composition of genetic resources, including through the application of biotechnology” [57]) are in many cases subject to regulations and control mechanisms on ethical and legal provenance based on international treaties. These include, for example, the “Washington Convention on International Trade in Endangered Species of Wild Fauna and Flora” [58] and the “Nagoya Protocol on Access to Genetic Resources and the Fair and Equitable Sharing of Benefits Arising from their Utilization (ABS) to the Convention on Biological Diversity” [59]. Compliance with these regulations includes strict administrative obligations and requires transparent traceability of the biological material. 

A regulated, clear, robust and accepted designation of animal cell material provides for traceability and is therefore indispensable for consistent scientific work and credible research results [3,9]. This necessity becomes particularly evident in the context of the long-term storage of living animal biosamples in wildlife cryobanks. Such continuously evolving archives aim to preserve valuable cellular and genetic material to preserve biodiversity [20,60,61,62,63] in the context of accelerated anthropogenic species extinction rates [64]. They further target promoting its broad application in different research fields and conservation efforts [65], comparative cell and development biology research [66], (advanced) assisted reproduction technologies ((a)ART) and stem-cell-associated techniques (SCAT) [1,24,67,68,69]. These biorepositories are in need of joint data and process standardization [2,19]. Informative unique identifiers will ease compliance tracing within the relevant legal frameworks of animal biosamples stored in cryobanks and exchanged internationally.

## 2. Methods

### 2.1. Requirements of a Standardized Nomenclature 

The developed standardized nomenclature was designed in consideration of the recommendations of the International Cell Line Authentication Committee (ICLAC) [9]. A standardized nomenclature must follow a formal pattern, i.e., a structured design characterized by predetermined rules, and be documented in a repository [11]. These nomenclatures should ideally generate human-readable names for easy recognition of the features of the material to be informative to human users. 

### 2.2. Nomenclature Components 

To establish a standardized and human-readable nomenclature for biological specimens which includes a wide range of species, it is imperative to take into account species name coding to contextualize the identifiers of the respective biosamples. Moreover, it is reasonable to include information on the biosample type, such as tissue or cell line. However, when including human-readable information on the species and the biosample, the number of characters in the identifier is likely to rapidly increase and even exceed a reasonable, intuitive and useful nomenclature length. Thus, a nomenclature design naturally faces a trade-off between information and length [3,4,11].

## 3. Results

### 3.1. Proposed Standardized Nomenclature—Defined Formal Pattern

**Figure 1:**
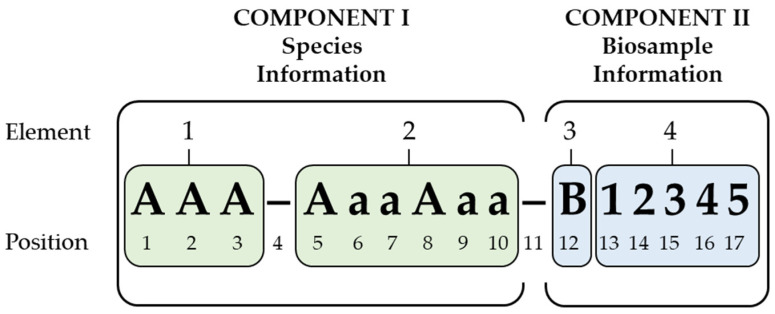
Schematic presentation of the nomenclature design with its components and elements. The 17-digit unique identifiers (UIs) are composed of four descriptive elements in a predefined formal pattern with distinct order 1–4, providing information on the species (component I) and the biosample (component II). The combination of elements 1 and 2 results in a robust species identifier. Each of the 17 positions is assigned a characteristic feature of upper case letter (positions 1, 2, 3, 5, 8 and 12), lower case letter (positions 6, 7, 9 and 10), hyphen (positions 4 and 11) or five 1-digit numbers between 0 and 9 (positions 13–17). Upper case letters indicate the first letter of a new word.

The present nomenclature design aims to provide a unique, stable and human-readable 17-digit alphanumerical identifier for viable animal biosamples at the species level. It follows a simple, predefined structure, which links two components: a unique and novel 10-digit alphabetical species code (component I), followed by a 1-digit prefix for biosample classification and a 5-digit ascending identification number for every new cellular biosample (component II) (see Table 2). The clarity and readability of the two components are strengthened by hyphens (see Figure 1). This design allows for 10^5^ possible standardized identifiers for each of the considered biosample types, tissue (T), cells (C), gametes (G) and embryos (E), within one species. It could be expanded for additional biosample types, such as multicellular models (M) (organoids, assembloids, blastoids, etc.).

### 3.2. Nomenclature Adaptation to Transformation Processes

**Figure 2:**
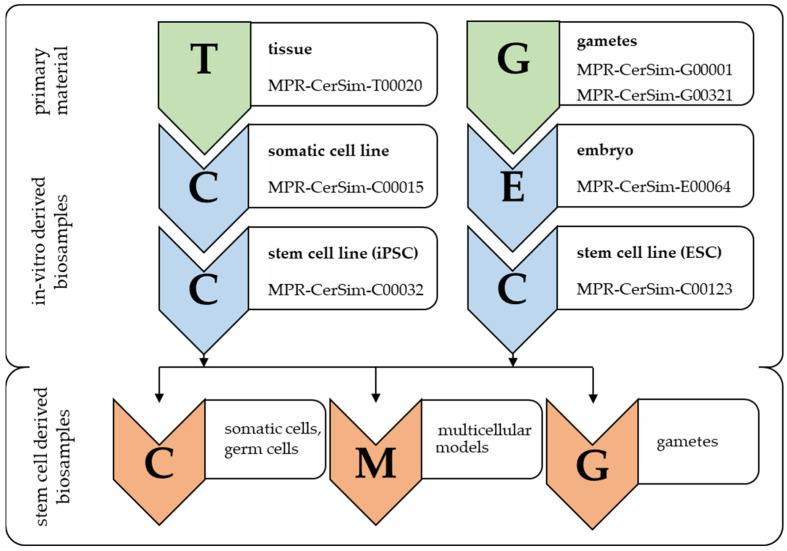
Alteration of biosample information coding (component II) according to downstream processing of biosamples. Example for White rhino (*Ceratotherium simum*) primary material. Left example: Skin tissue material of a White rhino, registered as, e.g., MPR-CerSim-T00020, is hypothetically processed into a somatic cell line (e.g., fibroblasts) and subsequently assigned a new UI, such as, e.g., MPR-CerSim-C00015. Aliquots of the latter are then reprogrammed into an iPSC line, and this new cell line is registered as, e.g., MPR-CerSim-C00032. Right example: Gametes of a White rhino, which are, e.g., assigned the UIs MPR-CerSim-G00001 and MPR-CerSim-G00321, are used for in-vitro fertilization, and one of the resulting embryos is assigned the UI MPR-CerSim-E00064. An ESC line, which would be derived from this embryo, is subsequently assigned a UI, e.g., MPR-CerSim-C00123. If stem-cell-derived biosamples such as somatic and germ cells, multicellular models or gametes are further developed, the prefix and subsequent number are then adjusted accordingly, e.g., to “M” or “G”.

Not integrated into such a nomenclature pattern is information such as derivation processes or hierarchy. Any transformation of a cellular biosample (e.g., genetic modifications) will therefore result in an individual, newly distributed identifier through the assignment of a new identification number and—if necessary—a new prefix (see Figure 2). A precise feature definition for every single position within the nomenclature (see Figure 1) prevents the possible confusion caused by ambiguous characters such as an upper case “O” and “I” or lower case “I” and the numbers 0 and 1. Examples of the nomenclature design are summarized in Table 3.

The naming scheme has been designed and is proposed in particular for the designation of the viable cellular material of non-domesticated species in order to set a standard for systematic referencing in expanding research on and with wildlife cells. Its applicability to the cell material of highly researched model species, such as mice, while maintaining human readability would require an extension of or change in the coding to cover high numbers of biosamples.

## 4. Discussion

### 4.1. Scheme for a Species Identifier

A universally valid coding structure that cyphers scientific species names does not exist to date. Species identifier codes are usually generated by different taxonomic databases at the subspecies level as either numerical or alphanumerical codes. Whereas, for example, the Catalogue of Life (COL) [70] generates an alphanumerical code (COL Identifier), different numerical codes are generated by the Integrated Taxonomic Information System (ITIS) [71] (Taxonomic Serial Number, TSN) and the NCBI taxonomy database [72] (Taxonomy ID, txid). As an example, for the species African elephant (*Loxodonta africana*), this results in the different codes “3W9KV” (COL Identifier), “584939” (ITIS TNS) and “9785” (NCBI txid). Including one of these predefined species codes in the biosample nomenclature would exclude other taxonomy databases. Moreover, the resulting nomenclature would ultimately be dependent on a unique species identifier, whose durability and stability are difficult to predict. We furthermore argue that the human readability of the species component in the nomenclature increases its acceptance and overall utility. Hence, the species coding we propose here for wildlife species refers to the International Code of Zoological Nomenclature. This binominal nomenclature is issued to provide a public and permanent scientific record and is defined as a combination of one generic name followed by one specific name, both containing two or more letters [73]. These official species names are globally applied by the scientific community to unambiguously describe animal species and, because of their human readability, are expected to be more stable than the aforementioned coding. The herein presented design for a universal, stable and human-readable alphabetical species identifier includes two interlinked elements: a 3-digit taxonomic classification (element 1), followed by a 6-digit acronym of the scientific binominal species name (element 2). The former serves as a prefix to the species name acronym and increases the unambiguity and strength of the species identifier. Cyphering remains at the species level, as sub-species are not considered. 

### 4.2. Adaptations and Limitations of the Nomenclature Design

The utility of a nomenclature to establish an identifier is measured by its ability to be adapted to new developments. For example, biosamples of reproductive, naturally occurring “inter-species hybrids”, as well as species with an exceptionally short binominal zoological name (see Supplementary Material Table S1), may not fit the proposed nomenclature pattern. However, such short scientific species names are extremely rare and only apply to one mammal species (Great evening bat (*Ia io*)), two fish species (Weedy cardinalfish (*Foa fo*) and *Betta pi*) and six invertebrate species [74]. Our proposed nomenclature design is flexible and well adjustable to these special cases (see Supplementary Material Table S1). Moreover, in the unlikely, although not impossible, event of any duplication of the 10-digit species code (component I), adjustments to the generic and specific acronyms can be made (element 2) (Table S1). Lastly, if a species is taxonomically not clearly assigned to a class, order and/or family but to a subdivision of these ranks, the closest assigned subdivision of the respective rank should be used (e.g., suborder instead of order) to create the taxonomic classification (element 1). 

In the infrequent event that a scientific species name is changed due to new scientific findings, the species identifier would need to be updated accordingly. Any resulting newly distributed identifiers for already named biosamples would have to be permanently linked to the outdated identifiers to maintain traceability. 

## 5. Conclusions

We herein propose a first, uniform, human-readable 17-digit alphanumerical nomenclature design that assigns standardized identifiers to animal-derived living cellular material such as tissues, cells, gametes, embryos and stem-cell-generated multicellular models. The predefined naming scheme is especially suggested for the designation of biosamples of wildlife species. It includes acronymized species and biosample information and allows simple adaptation according to the respective biosample type. Linking of the nomenclature-based name to a body of data which (i) uniformly characterizes the cellular material and its derivation, (ii) demonstrates the genealogy, sex and ID of the donor animal and (iii) evidences the legal and ethical provenance is indispensable to ensure clear reference to and the unambiguous traceability of the biomaterial, especially when it is published and transferred worldwide for research. A centralized repository of stable biosample names could provide such a resource and also allow for machine-based linking to other central registries, specifically RRIDs. Such a platform is thus required to make these persistent and the associated data publicly accessible (FAIR). Ideally, these unique identifiers will be automatically generated using an API by the centralized repository. We therefore emphasize the need for a centralized repository to associate the standardized biosample name with its metadata. Such collection, standardization and FAIRification of data are powerful tools to support the visibility and international exchange of valuable wildlife-derived biomaterial, thereby facilitating globally consistent scientific work in wildlife conservation and biomedical research.

## Figures and Tables

**Table 1:** Examples of assigned names for animal biosamples in absence of a standardized naming system.

	Biosample Name	Publication	Characterization
1	Snow leopard iPS	[45]	Snow leopard (*Panthera uncia*) iPSC line
2	J9F2	[46]	Japanese macaque (*Macaca fuscata fuscata*) iPSC line
3	RNA-iPSC #1	[47]	Common marmoset (*Callithrix jacchus*) iPSC lines
4	CM421F B-0-12 iPSC	[31]	
5	BWHGLi001	[48]	Naked mole rat (*Heterocephalus glaber*) iPSC line
6	ENL-2	[49]	Asian elephant (*Elephas maximus*) fibroblast lines
7	KCB 96008	[50]	
8	KDF	[51]	Sumatran rhinoceros (*Dicerorhinus sumatrensis*) fibroblast lines
9	SR-fibroblasts	[26]	
10	Fish 80	[52]	Rainbow trout (*Oncorhynchus mykiss*) tissues
11	pA03_wD06	[53]	
12	UCLAi090-A	[54]	Rhesus macaque (*Macaca mulatta*) iPSC line

**Table 2:** Summary and explanation of the nomenclature components and elements.

	Part	Structure	Explanation	Position
**COMPONENT I** **Species Information**	Element 1	AAA	A 3-digit acronym for taxonomic classification of the respective species formed by the first letter of class, order and family, all in upper case letters, e.g., MPC for all species within the family of Cercopithecidae (Old world monkeys): class: Mammalia, order: Primates, family: Cercopithecidae	1–3
	Element 2	AaaAaa	A 6-digit acronym of the binominal zoological nomenclature for the respective species as a sequence of the first three letters of its generic name, followed by the first three letters of its specific name in upper case and lower case letters, with each upper case letter indicating the first letter of the abbreviated words, e.g., MacMul for “*Macaca mulatta*” (Rhesus macaque)	5–10
**COMPONENT II** **Biosample Information**	Element 3	5 options for fixed letter	A 1-digit prefix of an upper case letter to specify the type of biosample as either tissue (T), cell line (C), gametes (G), embryo (E) or in vitro-generated multicellular model (M), (organoids, assembloids, blastoids/embryoids, etc.).	12
	Element 4	12345	A 5-digit ascending identification number between 00001 and 99,999, allowing for the distribution of 99,999 unique identifiers for the respective biosample type within one species	13–17

**Table 3:** Examples of the full nomenclature design. Summarized are combinations of diverse examples for component I (species information) and component II (biosample information).

				Full Nomenclature Design COMPONENTS I + II	
				Examples	Explanation
**COMPONENT II—Biosample Information**	primary material and biosamples directly derived thereof	Tissue —e.g., skin, blood	T	ACC-AndDiv-T00001	tissue biosample number 1 from a Chinese giant salamander (*Andrias davidianus*) with taxonomic assignment to Amphibia (class), Caudata (order), Cryptobranchidae (family)
		cell lines —e.g., somatic, stem, germ cells	C	APP-CyaSpi-C00125	cell biosample number 125 from a Spix’s macaw (*Cyanopsitta spixii*) with taxonomic assignment to Aves (class), Psittaciformes (order), Psittacidae (family)
		Gametes —i.e., spermatozoa and oocytes	G	MPR-DicSum-G00015	gamete biosample number 15 from the species Sumatran rhinoceros (*Dicerorhinus sumatrensis*) with taxonomic assignment to Mammalia (class), Perissodactyla (order), Rhinocerotidae (family)
		Embryos —in vivo- and in vitro-derived	E	MPR-CerSim-E00029	embryo number 29 of a White rhinoceros (*Ceratotherium simum*) with taxonomic assignment to Mammalia (class), Perissodactyla (order), Rhinocerotidae (family)
	stem-cell-derived	multicellular models—e.g., organoids, assembloids, blastoids	M	MPH-GorGor-M00012	multicellular model number 12 from the species Western gorilla (*Gorilla gorilla*) with taxonomic assignment to Mammalia (class), Primates (order), Hominidae (family)

## Data Availability

No new data were created or analyzed in this study. Data sharing is not applicable to this article.

## Supplementary Materials

The following supporting information can be downloaded at https://www.mdpi.com/article/10.3390/ani14111541/s1. Table S1: Summary of species acronym adjustments.

## References

[1] Hildebrandt T.B., Hermes R., Göritz F., Appeltant R., Colleoni S., de Mori B., Diecke S., Drukker M., Galli C., Hayashi K. (2021). The ART of bringing extinction to a freeze—History and future of species conservation, exemplified by rhinos. Theriogenology.

[2] Corrales C., Luciano S., Astrin J.J. (2023). Biodiversity biobanks: A landscape analysis. ARPHA Prepr..

[3] Luong M.X., Auerbach J., Crook J.M., Daheron L., Hei D., Lomax G., Loring J.F., Ludwig T., Schlaeger T.M., Smith K.P. (2011). A Call for Standardized Naming and Reporting of Human ESC and iPSC Lines. Cell Stem Cell.

[4] Kurtz A., Seltmann S., Bairoch A., Bittner M.S., Bruce K., Capes-Davis A., Clarke L., Crook J.M., Daheron L., Dewender J. (2018). A Standard Nomenclature for Referencing and Authentication of Pluripotent Stem Cells. Stem Cell Rep..

[5] Vasiliou V., Veselkov K., Bruford E., Reichardt J.K.V. (2021). Standardized nomenclature and open science in Human Genomics. Hum. Genom..

[6] Fujiyoshi K., Bruford E.A., Mroz P., Sims C.L., O’Leary T.J., Lo A.W.I., Chen N., Patel N.R., Patel K.P., Seliger B. (2021). Standardizing gene product nomenclature—A call to action. Proc. Natl. Acad. Sci. USA.

[7] McDonald A.G., Tipton K.F. (2023). Enzyme nomenclature and classification: The state of the art. FEBS J..

[8] Human Pluripotent Stem Cell Registry (hPSCreg). https://hpscreg.eu/.

[9] International Cell Line Authentication Committee (ICLAC) Naming a Cell Line, Essential Requirements. https://iclac.org/wp-content/uploads/ICLAC_Naming-a-Cell-Line_02-Mar-2023.pdf.

[10] Ludwig T.E., Andrews P.W., Barbaric I., Benvenisty N., Bhattacharyya A., Crook J.M., Daheron L.M., Draper J.S., Healy L.E., Huch M. (2023). ISSCR standards for the use of human stem cells in basic research. Stem Cell Rep..

[11] McMurry J.A., Juty N., Blomberg N., Burdett T., Conlin T., Conte N., Courtot M., Deck J., Dumontier M., Fellows D.K. (2017). Identifiers for the 21st century: How to design, provision, and reuse persistent identifiers to maximize utility and impact of life science data. PLoS Biol..

[12] Bandrowski A., Brush M., Grethe J.S., Haendel M.A., Kennedy D.N., Hill S., Hof P.R., Martone M.E., Pols M., Tan S.C. (2016). The Resource Identification Initiative: A cultural shift in publishing. J. Comp. Neurol..

[13] Research Resource Identification. https://www.rrids.org/.

[14] Cellosaurus. https://www.cellosaurus.org/.

[15] Bairoch A. (2018). The Cellosaurus, a Cell-Line Knowledge Resource. J. Biomol. Tech..

[16] BioSamples. http://www.ebi.ac.uk/biosamples.

[17] Courtot M., Cherubin L., Faulconbridge A., Vaughan D., Green M., Richardson D., Harrison P., Whetzel P.L., Parkinson H., Burdett T. (2019). BioSamples database: An updated sample metadata hub. Nucleic Acids Res..

[18] Courtot M., Gupta D., Liyanage I., Xu F., Burdett T. (2022). BioSamples database: FAIRer samples metadata to accelerate research data management. Nucleic Acids Res..

[19] Comizzoli P., Wildt D.E., Hainaut P., Vaught J., Zatloukal K., Pasterk M. (2017). Cryobanking Biomaterials from Wild Animal Species to Conserve Genes and Biodiversity: Relevance to Human Biobanking and Biomedical Research. Biobanking of Human Biospecimens.

[20] Ryder O.A., Onuma M. (2018). Viable Cell Culture Banking for Biodiversity Characterization and Conservation. Annu. Rev. Anim. Biosci..

[21] Ben-Nun I.F., Montague S.C., Houck M.L., Tran H.T., Garitaonandia I., Leonardo T.R., Wang Y.C., Charter S.J., Laurent L.C., Ryder O.A. (2011). Induced pluripotent stem cells from highly endangered species. Nat. Methods.

[22] Hildebrandt T.B., Hermes R., Colleoni S., Diecke S., Holtze S., Renfree M.B., Stejskal J., Hayashi K., Drukker M., Loi P. (2018). Embryos and embryonic stem cells from the white rhinoceros. Nat. Commun..

[23] Korody M.L., Ford S.M., Nguyen T.D., Pivaroff C.G., Valiente-Alandi I., Peterson S.E., Ryder O.A., Loring J.F. (2021). Rewinding Extinction in the Northern White Rhinoceros: Genetically Diverse Induced Pluripotent Stem Cell Bank for Genetic Rescue. Stem Cells Dev..

[24] Hayashi M., Zywitza V., Naitou Y., Hamazaki N., Goeritz F., Hermes R., Holtze S., Lazzari G., Galli C., Stejskal J. (2022). Robust induction of primordial germ cells of white rhinoceros on the brink of extinction. Sci. Adv..

[25] Zywitza V., Rusha E., Shaposhnikov D., Ruiz-Orera J., Telugu N., Rishko V., Hayashi M., Michel G., Wittler L., Stejskal J. (2022). Naïve-like pluripotency to pave the way for saving the northern white rhinoceros from extinction. Sci. Rep..

[26] Zywitza V., Frahm S., Krüger N., Weise A., Göritz F., Hermes R., Holtze S., Colleoni S., Galli C., Drukker M. (2022). Induced pluripotent stem cells and cerebral organoids from the critically endangered Sumatran rhinoceros. iScience.

[27] Ezashi T., Telugu B.P.V.L., Alexenko A.P., Sachdev S., Sinha S., Roberts R.M. (2009). Derivation of induced pluripotent stem cells from pig somatic cells. Proc. Natl. Acad. Sci. USA.

[28] Han X.P., Han J.Y., Ding F.R., Cao S.Y., Lim S.S., Dai Y.P., Zhang R., Zhang Y.R., Lim B., Li N. (2011). Generation of induced pluripotent stem cells from bovine embryonic fibroblast cells. Cell Res..

[29] Nagy K., Sung H.K., Zhang P.Z., Laflamme S., Vincent P., Agha-Mohammadi S., Woltjen K., Monetti C., Michael I.P., Smith L.C. (2011). Induced Pluripotent Stem Cell Lines Derived from Equine Fibroblasts. Stem Cell Rev. Rep..

[30] Liu J., Balehosur D., Murray B., Kelly J.M., Sumer H., Verma P.J. (2012). Generation and characterization of reprogrammed sheep induced pluripotent stem cells. Theriogenology.

[31] Yoshimatsu S., Nakajima M., Iguchi A., Sanosaka T., Sato T., Nakamura M., Nakajima R., Arai E., Ishikawa M., Imaizumi K. (2021). Non-viral Induction of Transgene-free iPSCs from Somatic Fibroblasts of Multiple Mammalian Species. Stem Cell Rep..

[32] Yoshimatsu S., Edamura K., Yoshii Y., Iguchi A., Kondo H., Shibuya H., Sato T., Shiozawa S., Okano H. (2021). Non-viral derivation of a transgene-free induced pluripotent stem cell line from a male beagle dog. Stem Cell Res..

[33] Li Z., Li Y., Zhang Q., Ge W., Zhang Y., Zhao X., Hu J., Yuan L., Zhang W. (2023). Establishment of Bactrian Camel Induced Pluripotent Stem Cells and Prediction of Their Unique Pluripotency Genes. Int. J. Mol. Sci..

[34] Takahashi K., Yamanaka S. (2006). Induction of pluripotent stem cells from mouse embryonic and adult fibroblast cultures by defined factors. Cell.

[35] Buehr M., Meek S., Blair K., Yang J., Ure J., Silva J., Mclay R., Hall J., Ying Q.L., Smith A. (2008). Capture of Authentic Embryonic Stem Cells from Rat Blastocysts. Cell.

[36] Liao J., Cui C., Chen S.Y., Ren J.T., Chen J.J., Gao Y., Li H., Jia N.N., Cheng L., Xiao H.S. (2009). Generation of Induced Pluripotent Stem Cell Lines from Adult Rat Cells. Cell Stem Cell.

[37] Miyawaki S., Kawamura Y., Oiwa Y., Shimizu A., Hachiya T., Bono H., Koya I., Okada Y., Kimura T., Tsuchiya Y. (2016). Tumour resistance in induced pluripotent stem cells derived from naked mole-rats. Nat. Commun..

[38] Yoshimatsu S., Sato T., Yamamoto M., Sasaki E., Nakajima M., Nakamura M., Shiozawa S., Noce T., Okano H. (2020). Generation of a male common marmoset embryonic stem cell line DSY127-BV8VT1 carrying double reporters specific for the germ cell linage using the CRISPR-Cas9 and PiggyBac transposase systems. Stem Cell Res..

[39] Yoshimatsu S., Murakami R., Sato T., Saeki T., Yamamoto M., Sasaki E., Noce T., Okano H. (2021). Generation of a common marmoset embryonic stem cell line CMES40-OC harboring a POU5F1 (OCT4)-2A-mCerulean3 knock-in reporter allele. Stem Cell Res..

[40] Simunovic M., Brivanlou A.H. (2017). Embryoids, organoids and gastruloids: New approaches to understanding embryogenesis. Development.

[41] Pasca S.P., Arlotta P., Bateup H.S., Camp J.G., Cappello S., Gage F.H., Knoblich J.A., Kriegstein A.R., Lancaster M.A., Ming G.L. (2022). A nomenclature consensus for nervous system organoids and assembloids. Nature.

[42] Rivron N.C., Frias-Aldeguer J., Vrij E.J., Boisset J.C., Korving J., Vivié J., Truckenmuller R.K., van Oudenaarden A., van Blitterswijk C.A., Geijsen N. (2018). Blastocyst-like structures generated solely from stem cells. Nature.

[43] Research Resource Identification Portal (RRID Portal). https://scicrunch.org/resources/.

[44] Usher M.B., Usher M.B. (1986). Wildlife conservation evaluation: Attributes, criteria and values. Wildlife Conservation Evaluation.

[45] Verma R., Holland M.K., Temple-Smith P., Verma P.J. (2012). Inducing pluripotency in somatic cells from the snow leopard (*Panthera uncia*), an endangered felid. Theriogenology.

[46] Nakai R., Ohnuki M., Kuroki K., Ito H., Hirai H., Kitajima R., Fujimoto T., Nakagawa M., Enard W., Imamura M. (2018). Derivation of induced pluripotent stem cells in Japanese macaque (*Macaca fuscata*). Sci. Rep..

[47] Nakajima M., Yoshimatsu S., Sato T., Nakamura M., Okahara J., Sasaki E., Shiozawa S., Okano H. (2019). Establishment of induced pluripotent stem cells from common marmoset fibroblasts by RNA-based reprogramming. Biochem. Biophys. Res. Commun..

[48] Lee S.G., Mikhalchenko A.E., Yim S.H., Lobanov A.V., Park J.K., Choi K.H., Bronson R.T., Lee C.K., Park T.J., Gladyshev V.N. (2017). Naked Mole Rat Induced Pluripotent Stem Cells and Their Contribution to Interspecific Chimera. Stem Cell Rep..

[49] Pavulraj S., Eschke K., Prahl A., Flugger M., Trimpert J., van den Doel P.B., Andreotti S., Kaessmeyer S., Osterrieder N., Azab W. (2019). Fatal elephant endotheliotropic herpesvirus infection of two young Asian elephants. Microorganisms.

[50] Chen Y.Z., Liu R.Q., Nai W.H., Yang M., He C.H., Bao Y.F. (1998). The Chromosome of Asian Elephant. Zool. Res..

[51] Jenuit M., Zainuddin Z.Z., Payne J., Ahmad A.H., Mat Yusof A., Md Isa M.L., Ibrahim M. (2021). Establishment and cryopreservation of fibroblast cell line from a Sumatran rhinoceros (*Dicerorhinus sumatrensis*). J. Sustain. Sci. Manag..

[52] BioSamples Database, Identifier SAMN27362692. https://www.ebi.ac.uk/biosamples/samples/SAMN27362692.

[53] BioSamples Database, Identifier SAMN13816208. https://www.ebi.ac.uk/biosamples/samples/SAMN13816208.

[54] Sosa E., Kim R., Rojas E.J., Hosohama L., Hennebold J.D., Orwig K.E., Clark A.T. (2017). An integration-free, virus-free rhesus macaque induced pluripotent stem cell line (riPSC90) from embryonic fibroblasts. Stem Cell Res..

[55] Wilkinson M.D., Dumontier M., Aalbersberg I.J., Appleton G., Axton M., Baak A., Blomberg N., Boiten J.W., Santos L.B.D., Bourne P.E. (2016). Comment: The FAIR Guiding Principles for scientific data management and stewardship. Sci. Data.

[56] Babic Z., Capes-Davis A., Martone M.E., Bairoch A., Ozyurt I.B., Gillespie T.H., Bandrowski A.E. (2019). Meta-Research: Incidences of problematic cell lines are lower in papers that use RRIDs to identify cell lines. eLife.

[57] European Commission Guidance Document on the Scope of Application and Core Obligations of Regulation (EU) No 511/2014 of the European Parliament and of the Council on the Compliance Measures for Users from the Nagoya Protocol on Access to Genetic Resources and the Fair and Equitable Sharing of Benefits Arising from Their Utilisation in the Union 2021/C 13/01. https://eur-lex.europa.eu/legal-content/EN/TXT/?toc=OJ%3AC%3A2021%3A013%3ATOC&uri=uriserv%3AOJ.C_.2021.013.01.0001.01.ENG.

[58] Convention on International Trade in Endangered Species of Wild Fauna and Flora (CITES) Text of the Convention 1973. https://cites.org/sites/default/files/eng/disc/CITES-Convention-EN.pdf.

[59] Secretariat of the Convention on Biological Diversity (2011). Nagoya Protocol on Access to Genetic Resources and the Fair and Equitable Sharing of Benefits Arising from Their Utilization to the Convention on Biological Diversity.

[60] Benirschke K. (1984). The Frozen Zoo Concept. Zoo Biol..

[61] Lermen D., Blömeke B., Browne R., Clarke A., Dyce P.W., Fixemer T., Fuhr G.R., Holt W.V., Jewgenow K., Lloyd R.E. (2009). Cryobanking of viable biomaterials: Implementation of new strategies for conservation purposes. Mol. Ecol..

[62] Saragusty J., Katkov I. (2012). Genome banking for vertebrates wildlife conservation. Current Frontiers in Cryobiology.

[63] Mooney A., Ryder O.A., Houck M.L., Staerk J., Conde D.A., Buckley Y.M. (2023). Maximizing the potential for living cell banks to contribute to global conservation priorities. Zoo Biol..

[64] Ceballos G., Ehrlich P.R., Barnosky A.D., García A., Pringle R.M., Palmer T.M. (2015). Accelerated modern human-induced species losses: Entering the sixth mass extinction. Sci. Adv..

[65] Angeles N.A.C., Catap E.S. (2023). Challenges on the Development of Biodiversity Biobanks: The Living Archives of Biodiversity. Biopreserv. Biobank..

[66] Lázaro J., Costanzo M., Sanaki-Matsumiya M., Girardot C., Hayashi M., Hayashi K., Diecke S., Hildebrandt T.B., Lazzari G., Wu J. (2023). A stem cell zoo uncovers intracellular scaling of developmental tempo across mammals. Cell Stem Cell.

[67] Saragusty J., Diecke S., Drukker M., Durrant B., Ben-Nun I.F., Galli C., Göritz F., Hayashi K., Hermes R., Holtze S. (2016). Rewinding the process of mammalian extinction. Zoo Biol..

[68] Herrick J.R. (2019). Assisted reproductive technologies for endangered species conservation: Developing sophisticated protocols with limited access to animals with unique reproductive mechanisms. Biol. Reprod..

[69] Bolton R.L., Mooney A., Pettit M.T., Bolton A.E., Morgan L., Drake G.J., Appeltant R., Walker S.L., Gillis J.D., Hvilsom C. (2022). Resurrecting biodiversity: Advanced assisted reproductive technologies and biobanking. Reprod. Fertil..

[70] Catalogue of Life (COL). https://www.catalogueoflife.org/.

[71] Integrated Taxonomic Information System (ITIS). https://itis.gov/.

[72] National Center for Biotechnology Information (NCBI) Taxonomy Database. https://www.ncbi.nlm.nih.gov/taxonomy.

[73] International Commission on Zoological Nomenclature (ICZN) (1999). International Code of Zoological Nomenclature.

[74] List of Short Species Names. https://www.wikiwand.com/en/List_of_short_species_names.

