# Supplementary material for: A Standardized Nomenclature Design for Systematic Referencing and Identification of Animal Cellular Material
Source: Animals (Basel). 2024 May 23;14(11):1541. doi: 10.3390/ani14111541 (PMC11171381; doi:10.3390/ani14111541)
Supplement: Supplementary file 1 [file animals-14-01541-s001.zip › animals-2963472-supplementary.pdf]

A Standardized Nomenclature Design for Systematic Referencing and Identification of Animal Cellular Material

Lisa Schrade, Nancy Mah, Anita Bandrowski, Ying Chen, Johannes Dewender, Sebastian Diecke, Christian Hiepen, Madeline A. Lancaster, Tomas Marques-Bonet, Sira Martinez, Sabine C. Mueller, Christopher Navara, Alessandro Prigione, Stefanie Seltmann, Jaroslaw Sochacki, Magdalena A. Sutcliffe, Vera Zywitza, Thomas B. Hildebrandt and Andreas Kurtz

Supplementary Material

Table S1. Summary of species acronym adjustments. The 6-digit species acronym (element 2) can be adjusted for (i) inter-species hybrids, (ii) exceptionally short scientific species names, and (iii) unforeseen conformity of a species code for different species. Adjustments only occur within the species acronym. The structure of the taxonomy acronym (element 1), prefix for biosample information (element 3) and identification number (element 4) remain the same.

Adjustment to	Structure	Explanation
Reference: Standard design	**AaaAaa**	A 6-digit acronym of the binominal zoological nomenclature for the respective species as a sequence of the first three letters of the generic name, followed by the first three letters of the specific name, in upper case and lower case letters, with each upper case letter indicating the first letter of the abbreviated words,<br>e.g., PanTro for "*Pan troglodytes*" (Common chimpanzee)
(i) Inter-species hybrids	**AaaAxA**	A 6-digit acronym of the binominal zoological nomenclature for naturally occurring inter-species hybrids as a sequence of the first three letters of the joint generic name, followed by the first letters of the specific names of both species, separated by an "x" indicating hybridization,<br>e.g., PanLxT for a hybrid between "*Panthera leo*" and "*Panthera tigris*" ("*Panthera leo* x *tigris*")
(ii) Short species names	**AaaAa^**<br>**Aa^Aaa**<br>**Aa^Aa^**	Adjustment of the 6-digit standard design for species names with generic and/ or specific names shorter than 3 digits. A 6-digit acronym (in UC and LC) according to the standard design, with gap-filling special character caret ^,<br>e.g., Ia^Io^ for "<u>*Ia*</u> <u>*io*</u>" (Great evening bat)
(iii) Ambiguous species code	**AAaaaa**<br>**AaAaaa**<br>**AaaaAa**<br>**AaaaaA**	Adjustment of the 6-digit standard design by shortening either the code for the generic or the specific name, with each upper case letter indicating the first letter of the new word
